# Invasive Mesquite (*Prosopis juliflora*), an Allergy and Health Challenge

**DOI:** 10.3390/plants9020141

**Published:** 2020-01-22

**Authors:** M. Iftikhar Hussain, Ross T. Shackleton, Ali El-Keblawy, María Del Mar Trigo Pérez, Luís González

**Affiliations:** 1 Research Institute of Science and Engineering, University of Sharjah, P.O. Box 27272, Sharjah 2141, UAE; 2 Plant Biology & Soil Science Department, Universidad de Vigo, 36310–Vigo (Pontevedra), Spain; luis@uvigo.es; 3 Centre for Invasion Biology, Department of Botany and Zoology, Natural Sciences Building, Private Bag X1, Stellenbosch University, Matieland 7600, South Africa; rtshackleton@gmail.com; 4 Institute of Geography and Sustainability, University of Lausanne, 1015 Lausanne, Switzerland; 5 Department of Applied Biology, College of Sciences, University of Sharjah, P.O. Box 27272, Sharjah 2141, UAE; akeblawy@sharjah.ac.ae; 6 Departamento de Biologia Vegetal, Universidad de Málaga, PO Box 59, 29080 Malaga, Spain; aerox@uma.es

**Keywords:** allergy, allergens, biological invasions, human heath, human well-being, pollens, prosopis, immunology, impact, invasive alien tree, invasive species

## Abstract

Mesquite (*Prosopis juliflora* (Sw.) DC), is an medium-sized tree (family Fabaceae, subfamily Mimosoideae), that has been intorcuded around the world. It is a noxious invasive species in Africa, Asia, and the Arabian Peninsula and a source of highly allergenic pollen in. The present article reviews the adverse allergenic effects of *P. juliflora* pollen on human and animal health. Several studies have diagnosed that allergenic pollens from *Prosopis* spp. can provoke respiratory problems. *Prosopis* pollen extracts have 16 allergenic components of which nine proteins were recognized as major allergens with some of them showing cross-reactivity. Clinically, understanding *Prosopis* pollen production, flowering seasonality, pollen load, and dispersal in the atmosphere are important to avoid allergic consequences for local inhabitants. Climate change and other pollution can also help to further facilitate allergenic issues. Furthermore, we document other human and animal health problems caused by invasive *Prosopis* trees. This includes flesh injuries, dental and gastric problems, and the facilitation of malaria. This review summarizes and enhances the existing knowledge about *Prosopis* flowering phenology, aeroallergen, and other human and animal health risks associated with this noxious plant.

## 1. Introduction

Many human activities, such as agriculture, recreation, global trade, and transportation have promoted both the intentional and accidental spread of species across their natural dispersal barriers [1,2]. A number of introduced species establish and spread becoming invasive in their new habitats, and can become serious ecological and health risks [3]. In particular, many invasive alien plant species have negative impacts on human and animal health and wellbeing through allergens, toxicity, and by causing injuries [4,5,6,7]. Understanding and documenting these effects on health are crucial to support management initiatives to reduce impacts. One such invasive alien genus that has serious human and animal health threats is *Prosopis* spp.

*Prosopis* L. is a genus with 44 species of medium-sized trees and shrubs belonging to family Fabaceae, subfamily Mimosoideae, with species occurring naturally in Africa, Asia, and North and South America [8]. *Prosopis* was found to occur as native or introduced species in 129 countries globally primarily in the world’s hot arid and semi-arid regions [6]. The numerous goods and services provided by *Prosopis* have led to global introductions and have made some species important for local communities [6,9]. At least 19 (invasive and weedy) of the 44 species in the genus *Prosopis* are known to generate benefits and costs in their introduced and invasive ranges, with the rest being primarily beneficial in their native ranges [6]. A particularly invasive species, *Prosopis juliflora,* has become widespread in many parts of the world. *Prosopis juliflora* is a fast-growing tree (5–10 m), native to frost-free tropical regions of Peru, Central America, and the Caribbean. It has long lateral shallow and deep taproot systems that are also able to fix nitrogen [6,8]. It has been introduced to different parts of the world for different purposes, but it has become an aggressive invader in most introduced ranges. *Prosopis juliflora* is now one of the most prominent of the invasive species from the genera, commonly invasding countries in Africa, the Middle East, and Asia, along with *Prosopis glandulosa, Prosopis velutina* and their hybrids in South Africa and Australia [6]. For example, it was introduced to Sudan in 1917 to combat desertification and to provide fuelwood [10]. Similarly, this species was brought to Lake Baringo, Kenya, in the 1980s to alleviate the fuelwood shortage [11,12]. In India, it was introduced during the late nineteenth century for the rehabilitation of sodic lands and to supply of fuelwood, fodder, timber, and fiber [13] and into Ethiopia in the 1970s and 1980s mainly for soil and water conservation [14]. This study, focus particularly on *P. juliflora* although the negative impacts mentioned here do occur for other invasive *Prosopis* species as well.This review examines current knowledge about the allergenic impact of *P. juliflora* on humans and associates it with the trees flowering phenology, pollen production, release, as well as other human and livestock health risks caused by the trees other traits, particularly, focusing on evidence from Africa and the Middle East. The review also highlights *Prosopis* aeroallergens, cross-reactivity, and its correlation with climate change consequences.

### History of P. juliflora in African and Arabian Peninsula

The history and range of distribution of the invasive *Prosopis* has been covered by several authors [15,16,17,18,19]. It is particularly prevalent as an invasive in Ethiopia, Kenya, Sudan, and South Africa [6]. According to Pasiecznik et al. [9], the *P. juliflora, P. pallida* complex, has been recorded in most of the African countries. However, *P. juliflora* is frost sensitive and severe frost can cause tree mortality in countries with cooler weather. Consequently, there is a doubt about the presence of this species in the Mediterranean climates of countries such as Morocco, Algeria, Tunisia, Libya, and Egypt. Similarly, Akrimi [18] stated that cold-sensitive *P. juliflora* was killed outright in Tunisia. Most of the Prosopis species that are present in these countries are more likely to be *P. glandulosa* Torr., *P. velutina* Wooton, *P. chilensis* (Molina) Stuntz, or hybrid forms from these species [8].

*Prosopis juliflora* has typically invaded areas in Africa and the Arabian Peninsula where water table is close to the ground surface, such as gravel deserts, open plains, sand sheets, rivers and flood plains, wadies, and edges of farms [19,20,21,22]. In Saudi Arabia, *P. juliflora* was reported as a ruderal plant, mostly found inside the urban and suburban areas but also other natural areas on the mainlands and islands of the region and is the most prominent invasive species in majority of lowlands [22]. *Prosopis juliflora* was introduced to the UAE in the 1970s for greening deserts [19,20,23]. During the last four decades, this species has escaped the planted forests and is currently considered a serious weed [23]. It has been reported in many natural habitats of UAE that have shallow water tables as well as in productive farmlands (Ali El-Keblawy, unpublished data). In Bahrain, scattered patches of young *P. juliflora* plants were recorded on coastal lowland, along with a huge single tree (said to be more than 500 years old and with more than 30 m tall) in the central plateau of the island. Similarly, this species was introduced to Qatar during the 1950s and it grows around farmland as well as in depressions [24].

## 2. Adverse Health Impacts of *Prosopis* in Humans

*Prosopis* invasions have a number of negative effects on human and animal health which impacts well-being and local livelihoods [4,6,7]. With regards to human health, there are evidences that *Prosopis* is inducing serious allergic reactions, even leading to septicemia. Furthermore, *Prosopis* can cause skin septicemia from thorn injuries and leads to an increased prevalence of malaria [24,25]. With regards to animal health, especially livestock, *Prosopis* is known to result in flesh injuries, and has negative gastro internal, neurological, as well as dental impacts which can make animals sick and even lead to death. We discuss this in more detail below.

### Prosopis Pollen, Allergy and Impact

**Figure 1:**
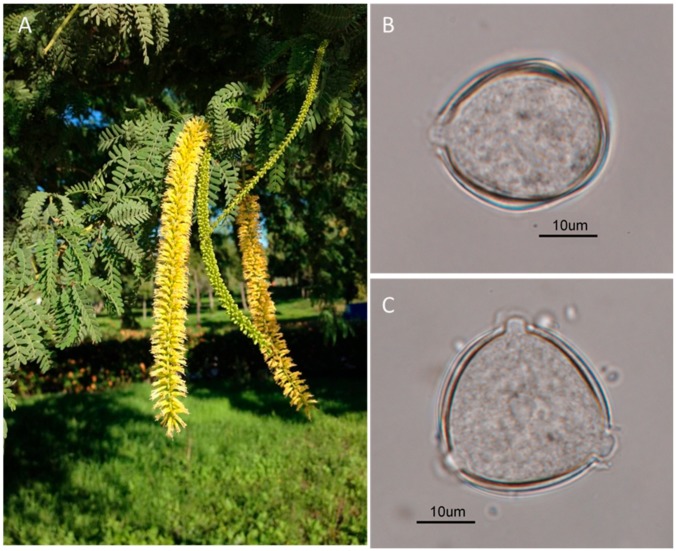
*P. juliflora:* plant showing the inflorescences (**A**) and pollen grains, equatorial (**B**) and polar (**C**) view, (light microscope, ×400, scale bar 10 μm).

Airborne *Prosopis* pollen grains are common where the tree is present and is a source of allergy reactions (pollinosis, rhinitis, conjunctivitis, asthma) in both its native and invasive range (Figure 1) [25]. Human sensitization to *Prosopis* was unveiled as early as the 1950s thanks to previous studies carried out by Ordman [26], Bieberdord and Swinny [27], Shivpuri and Parkash [28], Novey et al. [29], Thakur [30], and Lucas and Buckley [31], among others. Issues of allergies have also been reported for example, in the southwestern United States [29], in several regions of India [32], Pakistan [33], Saudi Arabia [34], South Africa [35,36], and a number of countries in the Neotropics/South America [37].

In the Middle East, *Prosopis* has been recognized as a major cause of allergic disease for some time, such as, in the Saudi Arabia [34,38], Kuwait [39], and United Arab Emirates [40], and high densities of invasion in urban areas exacerbate this [41]. Lab studies have concluded that *Prosopis* pollen is a serious threat to human health and has at least 13 different allergenic fractions [40]. In the UAE, about 45% of people tested were sensitive to *Prosopis* pollen (Figure 1) [29]. Al-Frayh et al. [34], studied the incidence of *P. juliflora* pollen in the population of four localities (Abha, Qassim, Hofuf, and Gizan) in Saudi Arabia by means of skin prick test and Radio allergosorbent-Test (RAST), for immediate hypersensitivity reaction to *Prosopis* allergens. Their results showed that 76.1% of patients in Qassim, 37.5% in Gizan, 29% in Abha, and 11% in Hofuf reacted positively to *Prosopis* antigens. Hight levels of *Prosopis* pollen grains were detected around Gizan,, exceeding 90 grains m^−3^ of air [34].

Al-Soqeer et al. [42], showed that 18 different allergen proteins and eight biogenic amines were detected in fifty *P. juliflora* genotypes collected from two regions of Saudi Arabia with different environmental conditions. The proteins had molecular weight ranging from 14 to 97 kDa. A list of different *P. juliflora* amine concentrations from the a-Qassim region of Saudi Arabia is demonstrated in Table 1, hilighting high levels of different allergy causing molecules for this invasie tree, but also veriations acorss different genotypes. Interestingly, they concluded from this study that environmental conditions, especially humidity, did not have any effect on allergen protein or their bioamine contents [42]. The impact of *P. juliflora* pollen extract was evaluated on respiratory allergy patients from Bikaner and Delhi, and it was found that *Prosopis* extract elicited positive skin reactions in 71 of the 220 patients [43]. They demonstrated the presence of 16 Immunoglobulin E (IgE) binding components in patient sera ranging from 24–95 kDa. Using gel filtration chromatography and polyacrylamide gel electrophoresis with 7.5% gels, Thakur and Sharma [44], separated *P. juliflora* into six fractions of 13, 20, 27.5, 41, 55.5, and 81 kd, and it was found that the 20 kd fraction had the highestallergenic activity.

Cross-racctivity an be a big issues regarding plant pollens. Cross-reactivity relates to the reaction between an antibody and an antigen that differs from the immunogen. The accurate knowledge about the cross-reactivity of *P. juliflora* pollen is crucial for diagnostics and the formulation of adequate immunotherapy. Cross-reactivity of *P. juliflora* pollen with other plants willfurther aggravate the health situation, sensitizing individuals with common proteins throughout the year. Dhyani et al. [45], has documented the cross-reactivity among *P. juliflora*, *Ailanthus excelsa,* and *Senna siamea* pollen components, and with *Holoptelea integrifolia*, *Putranjiva roxburghii,* and *Salvadora persica* pollen allergens. Similarly, More et al. [46] used 10–20% gels to investigate the allergens of *P. juliflora* in Arizona, USA, and reported Immunoglobulin E (IgE) responses to 59 and 66 kDa proteins in the pollen, wood and wood smoke of *P. multiflora.* The serum IgE binding to a 66 kDa allergen of *P. juliflora* pollen extract was significantly inhibited by lima bean seed extract and *P. juliflora* pollen extract. Purified proteins showed a close allergenic relationship with *A. excelsa*, *Senna siamea*, and *S. persica* tree pollens and with *Phaseolus lunatu*s. This cross-reactive purified proteins could, therefore, be used as a representative for multiple pollen/food allergens and may have implications for component-based therapy of allergies [47]. Assarehzadegan et al. [48], found, using SDS-PAGE, that there were several IgE-binding proteins in *P. juliflora* pollen extract with a molecular weight of 10–85 kDa. However, a molecular weight of approximately 20 and 66 kDa was the most frequent IgE reactive bands among the patient sera. On the other hand, inhibition experiments revealed high IgE cross-reactivity between *P. juliflora* and *Acacia farnesiana*. Thakur [49] reported a 45% success rate in using both the *P. juliflora* crude allergen extract and the 20 kDa glycoprotein fraction in human desensitization to *Prosopis*.

## 3. *Prosopis juliflora* Flowering Phenology and Pollen Attributes

A number of phenological factors contribute to the allergenic potential of *Prosopis* and need to be considered for aid in prevention and control, with pollen form flowers being a major issue. *Prosopis* flowers are small (4–6 mm long), arranged in inflorescences of various sizes and shapes, but generally in cylindrical spike-like racemes with 9.5–16.5 cm long and 300–400 flowers [50,51]. The inflorescences are arranged in clusters of 2–5 at the end of the branches [51]. *Prosopis* trees generally initiate flowering at a young age, in the third or fourth year after germination, but sometiems earlier. First flowering is dependent on optimal growth conditions and can be delayed under drought stress or when growing in very poor soils [9]. *Prosopis* species produce flowers every year increasing gradually for up to 10–20 years and can be expected to continue at high flowering levels for several decades.

Interestingly, in the native range, *P. juliflora* exhibits different physiological responses to environmental variables, mainly temperature, rainfall and day length [9], so variation in the onset of flowering is present between different populations due to climatic variation. Flowering is also variable within and between trees of the same population [9]. This is crucial to know when issuing precautionary advice to people. In its native range the *Prosopis juliflora* flowering season varies, with one or two periods of main flower production. It generally coincides with the wet season, from December to February and it is delayed from March to April with a second period from July to September.. Therefore, legume production generally overlaps with the end of the wet season improving seedling establishment or partly cover the dry season, ensuring pod consumption and seed dispersal by feral animals. In the invaded area *P. juliflora* present multiple different possibilities of flowering, associated not only to climatological conditions but to evolutionary associations, extremely fast, with pollinator insects. In Punjab (India) *P. juliflora* showed one flowering period from January to May with earlier flowering time period with the increase in the temperature [52,53], but in the ridge of Delhi (India) *P. juliflora* flowering occurs all year round reaching its peaks in April and September [30]. An almost continuous flowering, all-year-round, was observed in some invaded areas such as Brasil or Haiti [54]. This highlights the importance of seasonality when managing the tree to reduce pollen loads. Importance should be placed on controlling young trees before they flower out of season, but the need to focus on the removal older trees during flowering seasons to reduce pollen release and pollen loads. It also shows that awareness raising about health risks should coincide with flowering times, which differ acorss various invasive ranges.

### 3.1. Timing of Flowering and Pollen Production

There is controversy about time development of flowers in *P. juliflora*. Styles emerge from most flowers prior to anthesis and flowers remain in this state for some days [9]. Anthesis occurs when flowers are fully open and accessible to pollinators. Flower maturation is variable in the same tree, often lower flowers in the raceme are fully developed than the upper ones that are still immature (acropetal succession). Different maturation periods in the reproductive organs and flower development make *P. juliflora* plants self-incompatibles. Obligate outcrossing leading to high genetic variability as an evolutionary mechanism for survival in zones with a high variability in rainfall, temperature, and soil types. This trait, among others, allows to *P. juliflora* being an effective plant invader. Furthermore, long periods of asynchronous flower production lead to long periods of pollen release, which can prolong allergy issues.

### 3.2. Pollen Morphology, Dispersal Mechanisms and Diurnal Patterns

*Prosopis juliflora* presents tri-zono-colporate pollen grains, isopolar, and radio-symetric. Triangular in polar view, angulaperturate; elliptic in equatorial view, oblate. Medium size (P = 23 µm, E = 30 µm, non-acetolised). Apertures 3, composed by colpus + porus, situated in the equatorial zone. Exine 1.5–2 µm thickness with sexine lightly thicker than nexine. Tectum complete and infratectum columellar, with thin and hardly visible columellae with a light microscope. Surface psilate-perforated, lacking of supratectal elements. These characters are valuable in assisting the identification of taxa groups that can be used to differentiate between different species in the *Prosopis* genus and to identify tree pollens causing allergies. According to Sajjad et al. [55], the flowers of *P. juliflora* are self-incompatible, entomophilous, depending on insects for seed setting. This entomophilia is extremely generalized, with 77 insect visitors being reported in India. Hymenoptera (bees and wasps) and Diptera (flies) are the main visitors, attracted by the abundant pollen production and small quantities of concentrated nectar. *Prosopis juliflora* flowers exhibit exerted stamens, its pollen grains present a secondary anemophily and can easily pass into the atmosphere and become airborne. So, after gravitational deposition, pollens can deposit on many sufraces including the respiratory tract of humans thus causing allergies, but also in may other areas where they can later be disturbed and inhaled, as indicated in in fossil samples [39].

## 4. Impact of Climate Change on *P. juliflora* Pollen Allergy

Several factors including climate change are likely to affect pollen production, release and ultimately allergic disease [56]. Health professionals have shown that allergenic diseases will generally increase under climate change [57,58]. Due to higher atmospheric temperatures, local conditions in many areas will also become dryer which would facilitate the production of more pollens (e.g., for *Prosopis*), especially in arid and semi-arid areas. This can cause further negative consequences for the local inhabitants [59,60]. The patterns of sensitization to pollens depend on exposure risks which vary depending on the environmental characteristics (climate, geography, vegetation, etc.) of a given region, making predictions of allergy occurrence difficult. It is expected that climate change would exacerbate the prevailing stress in Arab Gulf countries [61].

CO_2_ is an important factor, but not the only one, affecting the abundance of species and their flowering under future climate change scenarios. The increase in CO_2_ will improve tree growth allowing them to develop faster andproduce more pollens and thus increasing the overall pollen load and the threat of allergies [62]. Plant species are expected to undertake spatial (poleward and upward) shifts in ranges [63] that will influence the abundance and distribution of allergenic plants and invasive species like *Prosopis* in particular [64,65]. Furthermore, several environmental pollutants, especially diesel engine exhaust particles, have been considered as significant pollen allergen releasing factors in the air. These particles contain minerals such as silica, iron, aluminum, magnesium, manganese, Sulphur, and others, which faciliates allergenicity. According to Knox et al. [66], pollen allergens associated with carbon particles from diesel engine fumes (DECP) would concentrate many allergic molecules in a single particle, and consequently, serious health risks might arise from this situation. Lately, to a lesser extent, but in addition to pollen exposure, the burning of *P. juliflora* wood will contibute to climate change, and its resulting smoke may be another source of exposure to some of these allergens [46,67].

## 5. Othere Imapcts of *Prosopis* for Human Health and Well-Being

### 5.1. Physical Injuries for Humans

There are a number of studies where communities highlight the *Prosopis* tree thorns that cause flesh related injuries, apparently resulting in some human fatalities [11,12,68]. *Prosopis* thorns are very hard and long, and when they pierce the skin they go deep into the flesh where the tip often breaks off. Deep pricks from *Prosopis* thorns are known to cause itching, and ounds can lead to lameness and amputation due to severe infection. For example, Mwangi and Swallow [12], describe how villages in Kenya are negatively impacted by “sharp, strong, and poisonous thorns of *Prosopis”.* This is also mirrored in a different region in Kenya [68], Eritrea [69], Ethiopia [70], South Africa [7], and Sudan [71].. There are records of hospitalization as a result of *Prosois* injuries and a few cases of death from infection [68]. Laxén [71], also reports cases of amputations due to infections as a result of *Prosopis* thorns in Sudan, with other similar complaints from other regions such as South Africa [6,7]. The *Prosopis* wax also causes irritation following contact with it [72].

### 5.2. Prosopis as Consequence of Increased Malaria

A community in Loboti, Kenya, reported the increased incidence of malaria associated with *Prosopis* invasions as the biggest negative impact induced by the invasive tree [11,12]. This has recently been verified by Muller et al. [73], who conducted experimental testing in Mali. *Prosopis* increases the incidence of malaria, as mosquitos feed primarily on tree sap (honeydew) and nectar. Females only need blood to reproduce otherwise they feed on plants and males only feed on plant products [73]. The invasion of *Prosopis*, therefore, provides more food for mosquitoes resulting in increased populations and consequently more vectors for carrying the malaria disease. The altered microhabitat (increase moisture in the leaf litter, and cooler areas) also might help with mosquito breeding and survival. In the study by Muller et al. [73], *Prosopis* was removed and resulted in 69.4% drop in mosquito population density. The proportion of sugar-fed female mosquitoes that potentially carry malaria dropped from 73% to 15% having obvious implications for human health.. Linked to these, Maundu et al. [68], highlight that communities reported an increased incidence of green biting flies is higher in areas with dense invasions, and further investigation is needed.

## 6. Adverse Health Impacts of *Prosopis* for Animals

### 6.1. Flesh Wounds

Like humans, livestock and wild animals also sustain flesh woods from *Prosopis* thorns which is considerd as a serious issue. It is particular problme when the tough thorns get lodged into the soft tissue in animal hooves sometimes making them lame [68]. The camels are particularly prone as they have soft feet [70]. Thorns have also been mentioned to damage livestock’s eyes causing blindness [74]. This impacts animal mobility and health, but also people’s livleihoods, partiualrly pastioralists in the arid regions of Africa.

### 6.2. Dental Impacts

Communities in Kenya took the government to court over a range of impacts *Prosopis* has had on their livelihoods, where a goat was famously brought in as a witness. One of these issues was the dental impacts livestock get as a result of eating *Prosopis* pods [12].. Livestock get disfigured jaws from chewing the hard *Prosopis* pods too frequently, often as a result of the tree displacing natural forage sources. In addition to this, the high sugar content in the pods results in tooth decay and loss, which in some cases leads to animals starving to death [11,12]. This is mirrored in Maundu et al. [68], where photos of dental impacts can also be seen. Similarly, the heavy consumption of pods by camels results in the loss of teeth affecting their overall feeding capacity and health [75].

### 6.3. Denervation Atrophy

When livestock consumes more than 20% *Prosopis* in their diet (which is common as it displaces native grazing species) they become prone to a disease called denervation atrophy [76]. Denervation atrophy is a motor neuron disease that impacts natural physiological functioning. This disease is locally known as “armeku” in Afar, Ethiopia, and has had a substantial negative effect on livestock health and thus local livelihoods in the area [77,78]. This has been confirmed by Silva et al. [79], who identified the presence of toxic alkaloids (juliprosopine and juliprosine) in the *Prosopis* leaves that can be responsible for neurotoxic damage in animals after consumption.

### 6.4. Digestive Effects

The cellulose present in the pods can cause serious problems to the animal’s digestive systems. In particular, overconsumption of pods can depress rumen bacterial cellulose activity and might induce a permanent impairment of digestion and eventualy mortality [80,81]. If eaten too much by cattle and goats *Prosopis* can result in diarrhea and sickness, as highlighted by local villagers in Kenya [68]. This is linked to the high tannin contents of *Prosopis* leaves [12,82,83]. It has also been documented that the consumption of *Prosopis* leaves by camels can cause diarrhea and sometimes constipation [82]. Furthermore, villagers in Kenya mentioned that *Prosopis* seeds get stuck within animal rumens resulting in their ill-health [68]. The seeds and their hard coating are not broken down well during digestion, and if too many pods are eaten they result in blockages and constipation. In Botswana there are reports of death of livestock after eating *Prosopis* most likely due to digestive tract issues [84].

## 7. Conclusions

Invasive *Prosopis juliflora* is a source of highly allergenic pollen grains which induces serious allergic reactions and asthma. Thorns cause flesh injuries and infections, and the high densities of invasive of trees, and the sap contained, leads to increased the prevalence of malaria. For animals, *Prosopis* causes flesh injuries, tooth decay, gastro internal issues, and neurological disorders all of which can lead to mortality. This can negatively affect farming communities’ livelihoods. We highlight that the health threats due to *P. juliflora* in the Arabian Peninsula and Africa has serious negative consequences. As a result of this evidence and other negative social-ecological effects caused by this invasive tree genus (see Shackleton et al., 2014) [6], control actions need to be better implemented at international, national, and regional levels to reduce theses negative effects. This is particularly important when one considers possible range shifts, changes in flowering phenology and increases in the amount of pollen production and allergenic potency, as well as increased invasion densities and competitive ability of *Prosopis* that could be brought about by changes in climate.

## Figures and Tables

**Table 1:** The list of amine concentrations in *Prosopis juliflora* genotypes from the Al-Qassim region, Saudi Arabia (Source: Al-Soqeer et al. [42]).

Genotypes	Tryptamine (µg/g)	β-phenylethy-lamine (µg/g)	Putrescine (µg/g)	Cadaverine (µg/g)	Histamine (µg/g)	Tyramine (µg/g)	Spermidine (µg/g)	Spermine (µg/g)
B1	54.4	45.6	45.6	7.4	18.6	132.2	17.5	4.4
B2	45.2	12.1	25.7	5.2	4.7	55.6	7.5	3.9
B3	83.3	47	126.3	79.5	37	396.2	49.5	7.4
B4	27.5	21.3	19.6	5.5	6.6	55.2	6.6	2.7
B5	61.5	38.3	66.2	9	17.7	174.1	24.8	5.6
B6	60.5	26	46.5	7.2	9.4	90.4	16.5	7.2
B7	11.4	4.3	5.5	10.3	1.3	13.7	1.6	0.2
B8	22	41.2	17.1	33.1	9.6	205.2	9	1.8
B9	35	36.8	98.6	4.6	30.2	154.1	92.5	30
B10	53.3	41.9	39.9	183.8	9.2	198.2	25.1	15.9
B11	24.1	15	18	4.8	8.3	63	4.4	1
B12	20.6	35.2	22.4	16.2	2.9	232	17.3	11.9
B13	37.5	34.2	39.8	7.4	9.2	191	24.7	6.9
B14	61.7	25.1	75.1	31	19.3	198.2	30.1	11.1
B15	46.7	40.5	63.7	39	7.7	224.6	45.5	12.4
B16	37.8	33.2	58.1	4.5	10.1	235.7	34.8	16.9
B17	28.1	20.8	54.3	77.7	11	213.1	36.9	15.7
B18	25.2	22.1	20.5	88.3	12.5	190	9.8	5.2
B19	27	21.8	32.5	93.6	15.5	247	22.5	13.5
B20	34.8	16.7	42.4	16.9	4.1	108.2	32.4	16.4
B21	40.8	11.7	24.8	6.7	7.9	112.6	6.8	6.5
B22	75.1	27.1	59.9	21.7	17.2	99.2	22.1	5.6
B23	9.1	4.6	6	0.83	0.9	25.9	3	4.1
B24	39.6	41.4	52.3	6.5	10.1	218	31.5	17
B25	12.6	6.2	13.9	4.6	4.3	58	8.1	5
S.E.*	±3.9	±3.1	±5	±8.6	±1.8	±17.4	±3.9	±1.3

S.E.* = Standard Error; difference between two means ≥ SE indicates significant difference.
